# Relationship of resilience, anxiety and injuries in footballers: Structural equations analysis

**DOI:** 10.1371/journal.pone.0207860

**Published:** 2018-11-26

**Authors:** Félix Zurita-Ortega, Ramón Chacón-Cuberos, Cristian Cofre-Bolados, Emily Knox, José Joaquín Muros

**Affiliations:** 1 Department of Didactics of Musical, Plastic and Corporal Expression, University of Granada, Granada, Spain; 2 Research Group HUM-238, University of Granada, Granada, Spain; 3 School of sciences of Physical Activity, Sport and Health ECIADES, University of Santiago, Santiago of Chile, Chile; 4 Sports Science and Physical Activity, University Santo Tomas, Santiago of Chile, Chile; 5 School of Health Sciences, The University of Nottingham, Nottingham, United Kingdom; University of Indianapolis, UNITED STATES

## Abstract

Resilience is a psychological characteristic which enhances personal assets and protects individuals from potential negative effects of various stressors. While this topic has been considered in the separate context of sports injuries and anxiety states, these issues have rarely been considered together. The objective of this study is to analyse the association between motivation to overcome injuries in football and the state of anxiety caused by those injuries. One hundred and eighty-five footballers from Spain were analysed by way of the Connor-Davidson Resilience Scale, the State-Trait Anxiety Inventory questionnaires, and an injury self-registration sheet. Statistical analyses were performed using structural equations. Results showed a direct and positive relationship between the capacity to face up to injuries or potential injuries and to adapt successfully to them for injured footballers, especially when anxiety was considered as a transitory emotional state. In addition, this relationship was stronger in non-injured sportspersons because their resilience capacity was not being impaired by the experience of an injury.

## Introduction

It is generally accepted that participation in professional sport carries an elevated risk of injury [1–2]. There are a series of factors which contribute to injury, much of the initial work on the psychology of injuries builds on the research by Andersen & Williams [3] such as age, inactivity, history of injuries [4] and fatigue [5]. Virtually all injuries can cause short or long periods away from undertaking the sporting specialism. There are also numerous studies which cite the incidence and prevalence of sports injuries in the context of organised sport and their relationship with some psychological attributes of players [4, 6–7]. Madzar et al. [8] and Zurita-Ortega et al. [9] have identified a relationship between injury occurrence and anxiety.

Anxiety is defined as a feeling of apprehension or threat that generates an increase in physiological activation [10]. This type of reaction is linked to the demands of the environment and especially in situations of competition in sport. This is due to external and internal judgments about individual capacity, which are perceived as a threat that generates insecurity. In the sports field, anxiety is an essential element of study, since it is closely related to sports performance and injury periods [11–12]. Specifically, Ensari et al. [13] defines two types of anxiety, highlighting anxiety-trait and anxiety-state. The first is related to chronic anxiety states linked to the personality of athletes, while the second is a temporary anxiety that appears due to specific circumstances that the athlete lives.

Resilience is a concept which has shown increasing importance in recent years within the scope of sports [14–16]. Although there are various definitions of resilience, most theories describe it as a psychological characteristic, which promotes positive adaptation when faced with adverse processes or periods of adversity [17–18]. Similarly, other authors have shown it to be an important element for improving recovery and reducing anxiety [19]. More current definitions have been provided by Windle [20] and Liu et al. [21], who conceptualize this construct as a process through which individuals use personal and environmental elements in order to redirect traumatic elements and stressors of everyday life. Thus, resilience capacity integrates cognitive and affective components with capacities and behaviours which allow healthy behaviours and positive responses to illness, disability and adversity to be developed [18–21].

Different dimensions which shape resilience as a psychosocial factor have been established. These include the locus of control and commitment (LCC), which determines how events are perceived and the strength of behaviour to overcome them. Other dimensions include the defiance of conduct oriented to the action (DCOA), self-efficacy and resistance to malaise (ARM), which are linked to the individual's ability to withstand harmful events and the behaviours executed. Finally, optimism and adaptation to stressful events (OASE), and spirituality (ES) have been highlighted as dimensions which assist emotional regulation of the problematic situation [15, 17–20].

Generally, resilience has been explored in various populations, including adolescents [18], older people [22] and migrants [23]. In the context of sport, there are very few studies which consider resilience. We note the studies of De la Vega et al. [24] relating to athletics, of Ruiz et al. [25] in the context of football, and most recently of Reche et al. [26] in judo in Spain. Outside of Spain, research has been conducted in athletes [15], Australian cricketers [12], Olympic champions [14], rugby players [16], American long distance runners [27], Chilean judokas [28] and top-level teams from various sporting disciplines [29]. Resilience is a highly complex concept influenced by a number of diverse individual, family and socio-cultural factors [30].

Anxiety and resilience have been widely studied in the sport context in relation to success and sport performance. For instance, Nezhad et al. [31] demonstrate the existence of a positive and direct relationship between resilience, well-being and sports success. In addition, several studies show the existence of a negative relationship between anxiety, resilience and performance [13, 31–32]. Nevertheless, these psychological factors have not been sufficiently studied in relation to the periods of injury experienced by athletes, especially in footballers because of their high competitive density.

Therefore, the general aim of this study was to analyse the association between the motivation of football players to overcome injury and the anxiety caused by that injury. The specific objectives were: (a) to identify the relationship between resilience and state-trait anxiety inventory scores (STAI-Trait) when a footballer is injured, and (b) to identify the relationship when the footballer is not injured.

## Materials and methods

### Design and participants

A descriptive and cross-sectional study was carried out with a single measure in a single group. A total of 185 male footballers, aged 15 to 34 (M = 21.21 years; SD = 4.90), participated in the study. Sample selection was by convenience using as exclusion criteria to play in teams of Spanish first and second division for professionals, to play in third division for semi-professionally and to play in categories under third division for amateur. Of the total sample, a 30.8% (n = 57) was professionally, a 10.3% (n = 19) was semi-professionally and the remaining 58.9% (n = 109) was amateur footballers.

### Instruments

The following variables were collected:

- Typology of Injury. Established via direct report of the injury status of the footballer at the time of data collection and labelled as ‘Injured’ or ‘Non Injured’, according to Fernández-García et al. [33].
- Anxiety State/Trait. The STAI Trait/State Questionnaire [34] was employed to assess the anxiety levels of the participants. This comprises 40 items that evaluate the degree of anxiety at a particular moment (state) and anxiety over a longer period of time (trait) through a four-point Likert-type scale. The present study produced a Cronbach Alpha of 0.67 and 0.72, for state and trait respectively, and 0.70 for the global scale.
- Resilience. The Connor-Davidson Resilience Scale (CD-RISC) proposed by Connor & Davidson [35] was used in order to estimate resilience. The CD-RISC includes 25 items evaluated using a four-point Likert scale ranging from 1 (strongly disagree) to 4 (strongly agree). It has been employed in a sporting context in Australia by Gucciardi et al. [6] and Ruiz et al. [25] in Spain. It is divided into the five dimensions set out above and produced a Cronbach´s alpha of 0.81, similar to that found in the original version (0.77). Additionally, the Cronbach Alpha for each indicator was 0.842 for locus of control and commitment (LCC), 0.803 for defiance of conduct oriented to the action (DCOA), 0.767 for self-efficacy and resistance to malaise (ARM), 0.794 for optimism and adaptation to stressful situations (OASE) and 0.665 for spirituality (ES).

### Procedure

Teams across Granada and Jaen provided a convenient sample for the researchers of the present study. Teams were sent a letter which briefly explained the objective of the study and invited them to take part. The letter detailed the voluntary nature of the investigation and detailed the written informed consent procedure. For participants aged under 18 years old, consent had to be signed by a legal guardian. Informed consent were obtained from all participants and from the parents of all participants under age 18. Questionnaires were administered to players during training sessions in October which is a period of the season characterised by a low requirement for performance and therefore a low trait anxiety. Further, measurements were made following a standard training session to prevent disruption to the athlete’s normal routine. Participants were informed that their answers would remain anonymous. There were no issues to report during data collection, following which, all footballers returned to their training routines. A total of 17 footballers were eliminated owing to their refusal to complete the questionnaires or due to them changing teams during the data collection period. The study was approved by the ethics committee of the university Santo Tomás (Chile) (CE UST Nº80/2014).

### Data analysis

Structural equation models of relationships between the measured constructs were developed using AMOS 21 to investigate the relationships between the variables analysed, all other analyses (normality analyses and descriptive statistics) were computed using SPSS 22.0. All data were assessed for normality by examining each measure’s Shapiro -Wilks test statistic.

Several authors recommend evaluation of goodness of fit to take into account various indices [36–38]. Chi-Square values associated to a non-significant *p*-value indicate good model fit [38]. Comparative fit index (CFI) values higher than 0.90 indicate acceptable model fit. Normalized fit index (NFI) values above 0.90 indicate good fit. Values of incremental fit index (IFI) above 0.90 indicate acceptable fit and values of the root mean square error of approximation (RMSEA) below 0.1 indicate good model fit [36, 39].

**Fig 1:**
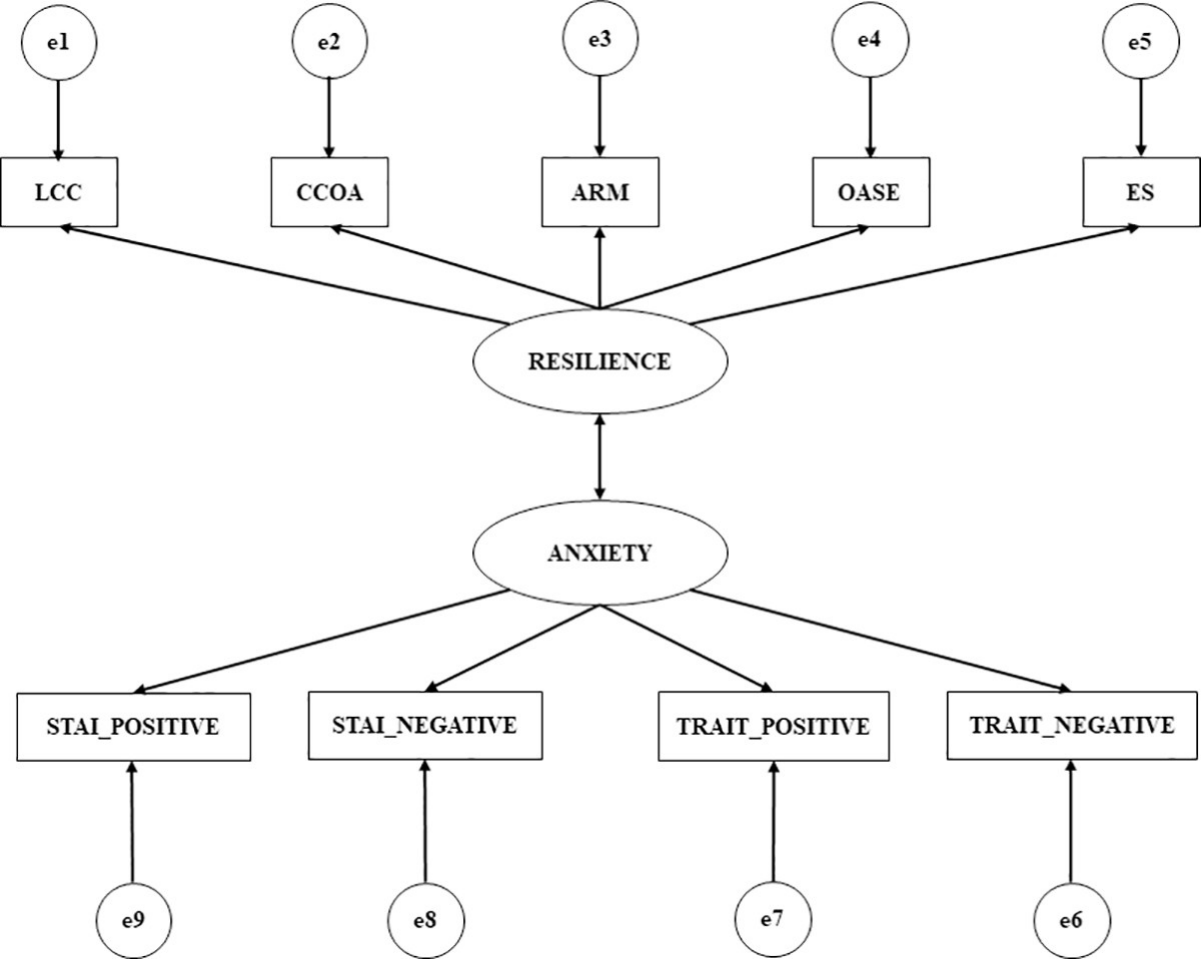
Model theorise: Resilience and anxiety. Note 1: LCC, locus of control and commitment; DCOA, defiance of conduct oriented to the action; ARM, self-efficacy and resistance to malaise; OASE, optimism and adaptation to stressful situations; and ES, spirituality.

In accordance with the objectives, the theoretical constructs predicted to influence resilience are considered in conjunction with the hypothetical model shown (Fig 1). This model, “Model theorise: Resilience and Anxiety”, is defined by two latent variables which are not measured directly but rather inferred through observable variables or indicators. The latent variable ‘resilience’ is inferred from five indicators: locus of control and commitment (LCC); defiance of conduct oriented to the action (DCOA); self-efficacy and resistance to malaise (ARM); optimism and adaptation to stressful situations (OASE) and spirituality (ES). Anxiety constitutes an exogenous latent variable and is inferred from four indicators, two of which relate to state anxiety and two of which relate to trait anxiety. All indicators can be expressed in a positive or negative direction. What is more, the measure takes into account the reliability of the measurements by incorporating the measurement errors into the model, making it possible to control them directly.

## Results

A structural model was designed to estimate the relationships between the measured constructs. Model fit is evaluated to ascertain the compatibility between the model proposed and the empirical evidence obtained. The resultant Chi-square value was associated with *p* and was significant (χ2 = 58.37; df = 26; *p* = 0.001) although we must take into account that this statistic, as an index, does not have an upper limit, for which reason it cannot be interpreted in a standardised manner. Thus, other standardised fit indexes that are less sensitive to the sample size are shown.

The model demonstrated acceptable fit to the data using the goodness of fit index (GFI = 0.93), the comparative fit index (CFI = 0.93), the incremental fit index (IFI = 0.93), the adjusted goodness of fit index (AGFI = 0.88) and RMSEA, which produced a value of 0.06.

Emanating from the analysis done on resilience and anxiety theoretical model, we observed the direct and indirect unidirectional and bidirectional relationships between these constructs through a multi-group analysis of the moderating effect among injured and non-injured footballers. In the theoretical model we can observe the structural analysis incorporating the correlation/covariance between the latent variables (Resilience and Anxiety), identified by way of bidirectional arrows, and the causality relationships among them and the observed variables or indicators which are marked with unidirectional arrows.

The results presented in Table 1 and Table 2 display the estimate, standard error (SE) and critical ratios (CR). The CR is calculated using the quotient between the estimate of the parameter and its corresponding SE. With respect to the footballers who did not experience a minor or serious injury, we can see that the regression weights of the indicators with respect to their latent variables are statistically significant.

**Table 1:** Weights and standardized regression weights in injured players.

Relationship between variables			P.R.				P.E.R.
			Estimations	S.E.	C.R.	*P*	Estimations
LCC	←	RESILIENCE	1.000	-	-	-	0.904
DCOA	←	RESILIENCE	0.368	0.114	3.230	***	0.286
ARM	←	RESILIENCE	0.605	0.070	8.630	***	0.681
OASE	←	RESILIENCE	0.851	0.078	10.862	***	0.845
ES	←	RESILIENCE	0.404	0.131	3.092	-	0.275
TRAIT_N	←	STAI_TRAIT	1.000	-	-	-	0.680
TRAIT_P	←	STAI_TRAIT	-0.929	0.158	-5.895	***	-0.629
STAI_N	←	STAI_TRAIT	1.114	0.170	6.553	***	0.761
STAI_P	←	STAI_TRAIT	-0.918	0.157	-5.866	***	-0.625
RESILENCE	↔	STAI_TRAIT	0.049	0.015	3.285	***	0.378

P.R., Regression Weights; P.E.R., Standardized Regression Weights; S.E., Estimation of error; C.R., Critical Ratio.

LCC, locus of control and commitment; DCOA, defiance of conduct oriented to the action; ARM, self-efficacy and resistance to malaise; OASE, optimism and adaptation to stressful situations; and ES, spirituality.

*** *p* < 0.001.

**Table 2:** Weights and standardized regression weights in non-injured players.

Relationship between variables			P.R.				P.E.R.
			Estimations	S.E.	C.R.	*p*	Estimations
LCC	←	RESILIENCE	1.000	-	-	-	0.797
DCOA	←	RESILIENCE	0.569	0.214	2.657	-	0.395
ARM	←	RESILIENCE	0.819	0.122	6.703	***	0.872
OASE	←	RESILIENCE	1.049	0.150	7.004	***	0.916
ES	←	RESILIENCE	0.590	0.243	2.430	-	0.363
TRAIT_N	←	STAI_TRAIT	1.000	-	-	-	0.719
TRAIT _P	←	STAI_TRAIT	-0.620	0.249	-2.489	-	-0.400
STAI_N	←	STAI_TRAIT	1.140	0.263	4.340	***	0.883
STAI _P	←	STAI_TRAIT	-0.565	0.218	-2.590	-	-0.416
RESILENCE	↔	STAI_TRAIT	0.059	0.022	2.671	***	0.609

P.R., Regression Weights; P.E.R., Standardized Regression Weights; S.E., Estimation of error; C.R., Critical Ratio.

LCC, locus of control and commitment; DCOA, defiance of conduct oriented to the action; ARM, self-efficacy and resistance to malaise; OASE, optimism and adaptation to stressful situations; and ES, spirituality.

*** *p* < 0.001.

**Fig 2:**
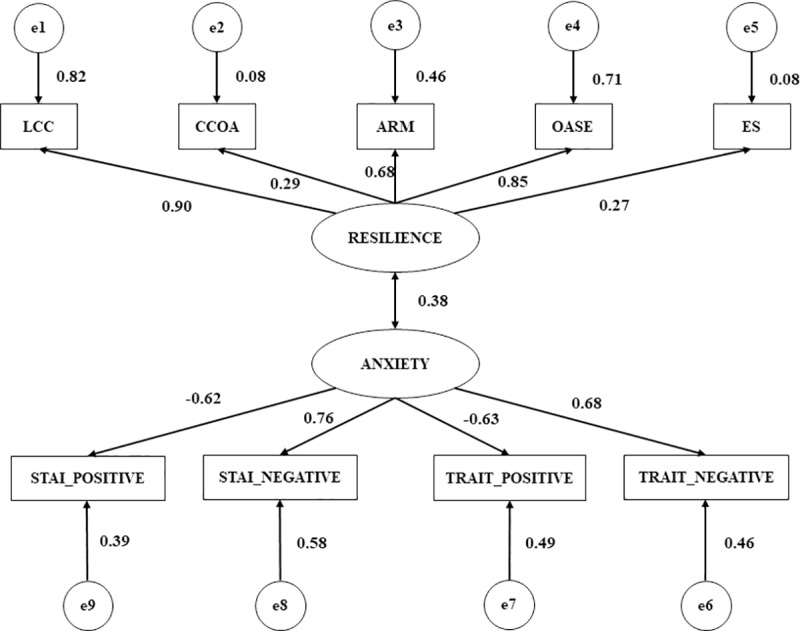
Multigroup structural equation model: Injured. Note 1: LCC, locus of control and commitment; DCOA, defiance of conduct oriented to the action; ARM, self-efficacy and resistance to malaise; OASE, optimism and adaptation to stressful situations; and ES, spirituality.

In Table 1 we can observe the estimate of the parameters of the model pertaining to the injured footballers. The loadings of the indicators relating to both latent variables (Resilience and Anxiety) are significant (*p* < 0.001), as the correlation between these two. This states that for injured footballers there is a direct and positive relationship between the capacity to accept (i.e. face up to) injuries and to be able to adapt successfully (r = 0.38), which is shown via a transient emotional state using the STAI Trait/State Questionnaire (Fig 2).

In addition, significant relationships were observed among all categories of resilience apart from spirituality and its global construct (*p* < 0.001). All relationships were direct and positive, and the greatest correlation strength was shown for LCC (r = 0.90), followed by OASE (r = 0.85) and ARM (r = 0.98). CCOA demonstrated the weakest relationship (r = 0.27). Furthermore, significant associations were shown for all categories of anxiety in injured footballers (*p* < 0.001). In this case, Stai-Positive and Trait-Positive were negatively related to anxiety (r = -0.62; r = -0.63, respectively) while Stai-Negative and Trait-Negative were positively related to anxiety (r = 0.76; r = 0.68, respectively).

**Fig 3:**
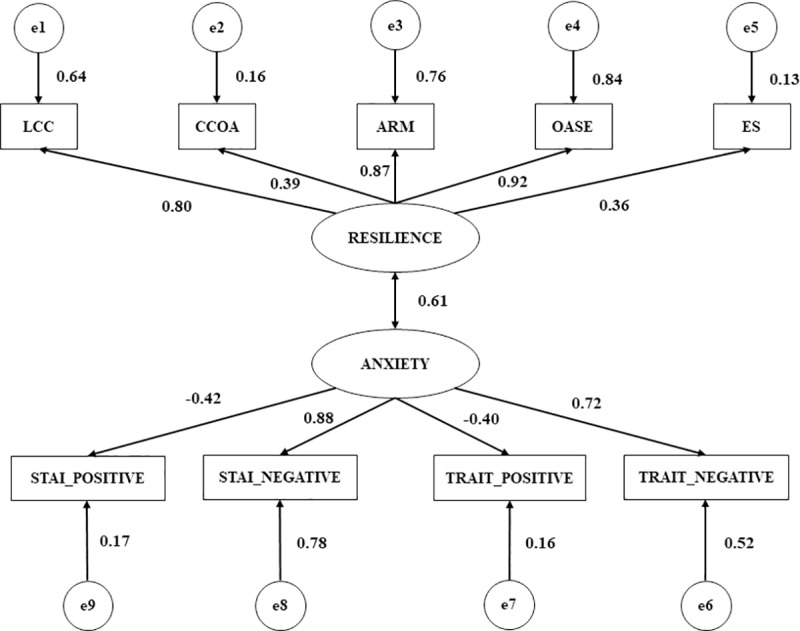
Multigroup structural equation model: Non-injured. Note 1: LCC, locus of control and commitment; DCOA, defiance of conduct oriented to the action; ARM, self-efficacy and resistance to malaise; OASE, optimism and adaptation to stressful situations; and ES, spirituality.

In Table 2 we can observe the estimate of the parameters of the model pertaining to non-injured footballers (Fig 3). The loadings of the indicators relating to global resilience (*p* < 0.001), ARM (r = 0.87) and OASE (r = 0.84) were significant. In addition, there was a positive correlation among resilience and Stai-Trait (r = 0.61) which was significant at level of *p* < 0.001. This signifies that for non-injured footballers there was a direct and positive relationship between the capacity to face up to potential injuries and the ability to adapt successfully should an injury be sustained in the future (r = 0.61). This relationship was stronger for non-injured footballers than for injured footballers. Moreover, a significant relationship was shown for Stai-Negative and Stai-Trait in non-injured footballers (*p* < 0.001). In this case, this association was direct and positive (r = 0.88).

## Discussion

The findings of the present study reveal a relationship between the motivation of footballers to overcome injuries and the state of anxiety resulting from such injuries. A number of previous studies have examined aspects of football from various perspectives in the scientific field -sociological, technical, tactical and performance- [1, 4, 40]. There have been consistent efforts in recent years to determine the causality of a variety of types of football injuries and establish how to deal with them effectively. Readers are directed to relevant papers that discuss diverse traumatic injuries and less common injuries sustained in the competitive environment by both male and female athletes [41–44]. The present study has identified the potential importance of resilience as an important element within the context of injuries in football.

We have discussed two models in relation to injured footballers. Resilience was directly correlated with the values obtained from the anxiety questionnaire. This suggests that injured footballers experience a state of anxiety and concern as a direct result of sustaining an injury. This is supported by previous research conducted by Liberal et al. [45] and Fernández-García et al. [33]. There is also some evidence that during periods of competition, when the footballer is most determined to compete but is prevented from doing so by injury, subsequent feelings of anxiety could actually prolong the duration of the injury [46–47]. It is important to note that all measurements were made at the beginning of the season, meaning that state anxiety levels could have been lower than expected during periods of high competition, and this may influence the strength of the correlations identified for resilience [45, 48].

It was observed that the capacity of resilience and anxiety in sport were positively related showing higher correlations in non-injured athletes. It is possible that the capacity for resilience of non-injured athletes is higher because it has not been diminished by the frustration experienced by injured athletes generated when they are not able to compete [7, 16]. In addition, the defiance of conduct oriented to the action, which most closely describes this, did not demonstrate significant associations in non-injured athletes, supporting that previous idea. Likewise, it was obtained a greater influence of state and trait anxiety in the global levels of anxiety of injured athletes, revealing how this can be determined by their own psychological conditions and contextual factors Zurita-Ortega et al. [9].

It is also important to consider resilience during periods of injury as this psychological characteristic that can promote a much more positive adaptation to adverse processes or periods of adversity, such as suffering an injury [17–18]. In this line, Connor et al. [35] have stated that improving resilience can bring about more positive states of anxiety and accelerate the rehabilitation process. Resilience is also a highly desirable characteristic for athletes to have in sport given the stressors and challenges that they encounter [14, 29]. The locus of control is often the strongest indicator for resilience in injured players, revealing the importance of positive perceptions that on is adequately overcoming an injury event and capable of maintaining a long-term improvement behavior [19–20, 35].

With regards to footballers who are not injured and therefore, have no conscious preoccupation with an adverse and performance-restricting consequence, there was a stronger correlation between resilience and anxiety. This is consistent with the work of Olmedilla et al. [48] who found that the tension levels of a sportsperson affect all areas of his or her activity, including their sport performance. An uninjured sportsperson will therefore not experience the internal conflict capable of diminishing their capacity of resilience. Specifically, the strongest indicator of resilience in non-injured athletes was optimism, which aligns with findings of Galli et al [29], who established that the perception of achievement in sports practice is not impaired by anxiety generated by the injury.

Considering the potential impact of sports injuries on a sportsperson and the wider sport, it would be appropriate and advisable to put into action injury prevention programmes adapted to each individual [9]. In this regard, some previous studies have introduced psychological interventions to tackle recovery from injury and to prevent subsequent sports injuries from occurring [48–50]. In addition, it would be useful to develop programs directed at improving resilience capacity with a view to reducing anxiety during the period of injury. Specifically, locus of control and commitment, optimism, and the ability to adapt to stressful situations should be targeted as key dimensions. This has the potential to reduce the recovery period and the likelihood of relapses in addition to increasing motivation during challenging events [51–52].

Finally, it is important to highlight some of the limitations of the present study which could influence interpretation of the findings. Firstly, the sample size was relatively small and so a greater number of sportspersons need to be recruited to confirm these preliminary findings. The research should also be extended to a greater variety of sports including coactive sports to enable meaningful comparison of results. Another limitation is the timing of measurements which were taken during a period of low performance burden. It would be interesting to take measurements at various times of the season in order to observe fluctuations of state anxiety and its relationship with resilience. Finally, the present study included only males it would be interesting to carry out subsequent studies on females to enable further interesting comparisons.

## Conclusions

The model developed to observe the relationships between motivation to overcome injuries in footballers and the state of anxiety provoked by the sustained injury satisfactorily fits the empirical data. In injured footballers, there is a direct and positive relationship between the capacity to face up to injuries and to successfully adapt to them (i.e. resilience) and state anxiety (i.e., anxiety as a transitory emotional state). This relationship was stronger in uninjured sportspersons because their resilience capacity was not being impaired by the stresses of a period of injury. With regards to resilience, the defiance of behaviour was significant in injured players but was not significant for non-injured players. In addition, negative state anxiety was the only factor associated with global anxiety in non-injured players, while all categories of anxiety were related to the global dimensions in injured players.

## Supporting information

S1 Fig Model theories: Resilience and Anxiety. Note 1: LCC, locus of control and commitment; DCOA, defiance of conduct oriented to the action; ARM, self-efficacy and resistance to malaise; OASE, optimism and adaptation to stressful situations; and ES, spirituality.
(DOCX)

S2 Fig Multigroup structural equation model: Injured. Note 1: LCC, locus of control and commitment; DCOA, defiance of conduct oriented to the action; ARM, self-efficacy and resistance to malaise; OASE, optimism and adaptation to stressful situations; and ES, spirituality.
(DOCX)

S3 Fig Multigroup structural equation model: Non-injured. Note 1: LCC, locus of control and commitment; DCOA, defiance of conduct oriented to the action; ARM, self-efficacy and resistance to malaise; OASE, optimism and adaptation to stressful situations; and ES, spirituality.
(DOCX)

S1 Table Weights and standardized regression weights in injured players. Note 1: P.R., Regression Weights; P.E.R., Standardized Regression Weights; S.E., Estimation of error; C.R., Critical Ratio. Note 2: LCC, locus of control and commitment; DCOA, defiance of conduct oriented to the action; ARM, self-efficacy and resistance to malaise; OASE, optimism and adaptation to stressful situations; and ES, spirituality. Note 3: *** p < 0.001.
(DOCX)

S2 Table Weights and standardized regression weights in non-injured players. Note 1: P.R., Regression Weights; P.E.R., Standardized Regression Weights; S.E., Estimation of error; C.R., Critical Ratio. Note 2: LCC, locus of control and commitment; DCOA, defiance of conduct oriented to the action; ARM, self-efficacy and resistance to malaise; OASE, optimism and adaptation to stressful situations; and ES, spirituality. Note 3: *** p < 0.001.
(DOCX)
