# Correction: Relationship of resilience, anxiety and injuries in footballers: Structural equations analysis

**DOI:** 10.1371/journal.pone.0212083

**Published:** 2019-02-07

**Authors:** Félix Zurita-Ortega, Ramón Chacón-Cuberos, Cristian Cofre-Bolados, Emily Knox, José Joaquín Muros

There are errors in the Reference list.

Reference 11 is incorrect. The correct reference is: Johnson U, Tranaeus U, Ivarsson A. Current status and future challenges in psychological research of sport injury prediction and prevention: A methodological perspective. Rev Psicol Deport 2014; 23: 401–409.

Reference 24 is incorrect. The correct reference is: De la Vega R, Rivera O, Ruiz R. Hardiness in endurance races: a comparison between skyrunning and 10 kilometers. Rev Psicol Deport 2011; 20: 445–454.

Reference 25 is incorrect. The correct reference is: Ruiz R, De la Vega R, Poveda J, Rosado A, Serpa S. Psychometric analysis of the resilience scale in the sport of football. Rev Psicol Deport 2012; 21: 143–151.

Reference 45 is incorrect. The correct reference is: Liberal R, Escudero JT, Cantallops J, Ponseti J. Psychological impact of sports injuries related to psychological well-being and the anxiety associated to competitive sports. Rev Psicol Deport 2014; 23: 451–456.

Reference 47 is incorrect. The correct reference is: Fernandes HM, Machado-Reis V, Vilaça-Alves J, Saavedra F, Aidar FJ, Brustad R. Social support and sport injury recovery: An overview of empirical findings and practical implications. Rev Psicol Deport 2014; 23: 445–449.
