# Supplementary material for: Relationship of resilience, anxiety and injuries in footballers: Structural equations analysis
Source: PLoS One. 2018 Nov 26;13(11):e0207860. doi: 10.1371/journal.pone.0207860 (PMC6257929; doi:10.1371/journal.pone.0207860)
Supplement: S2 Fig — Note 1: LCC, locus of control and commitment; DCOA, defiance of conduct oriented to the action; ARM, self-efficacy and resistance to malaise; OASE, optimism and adaptation to stressful situations; and ES, spirituality. (DOCX) [file pone.0207860.s002.docx]

**Supporting information**

**S2 Fig. Multigroup structural equation model: injured**. Note 1: LCC, locus of control and commitment; DCOA, defiance of conduct oriented to the action; ARM, self-efficacy and resistance to malaise; OASE, optimism and adaptation to stressful situations; and ES, spirituality.
