# Supplementary material for: Relationship of resilience, anxiety and injuries in footballers: Structural equations analysis
Source: PLoS One. 2018 Nov 26;13(11):e0207860. doi: 10.1371/journal.pone.0207860 (PMC6257929; doi:10.1371/journal.pone.0207860)
Supplement: S2 Table — Note 1: P.R., Regression Weights; P.E.R., Standardized Regression Weights; S.E., Estimation of error; C.R., Critical Ratio. Note 2: LCC, locus of control and commitment; DCOA, defiance of conduct oriented to the action; ARM, self-efficacy and resistance to malaise; OASE, optimism and adaptation to stressful situations; and ES, spirituality. Note 3: *** p < 0.001. (DOCX) [file pone.0207860.s005.docx]

**Supporting information**

**S2 Table. Weights and standardized regression weights in non-injured players.** Note 1: P.R., Regression Weights; P.E.R., Standardized Regression Weights; S.E., Estimation of error; C.R., Critical Ratio. Note 2: LCC, locus of control and commitment; DCOA, defiance of conduct oriented to the action; ARM, self-efficacy and resistance to malaise; OASE, optimism and adaptation to stressful situations; and ES, spirituality. Note 3: *** p < 0.001.
